# Neurological Manifestations of SARS-CoV-2 Infection: Protocol for a Sub-analysis of the COVID-19 Critical Care Consortium Observational Study

**DOI:** 10.3389/fmed.2022.930217

**Published:** 2022-07-22

**Authors:** Denise Battaglini, Lavienraj Premraj, Matthew Griffee, Samuel Huth, Jonathon Fanning, Glenn Whitman, Diego Bastos Porto, Rakesh Arora, Lucian Durham, Eric Gnall, Marcelo Amato, Virginie Williams, Alexandre Noel, Sabrina Araujo De Franca, Gordan Samoukovic, Bambang Pujo, David Kent, Eva Marwali, Abdulrahman Al-Fares, Stephanie-Susanne Stecher, Mauro Panigada, Marco Giani, Giuseppe Foti, Paolo Pelosi, Antonio Pesenti, Nicole Marie White, Gianluigi Li Bassi, Jacky Suen, John F. Fraser, Chiara Robba, Sung-Min Cho

**Affiliations:** ^1^Anesthesia and Intensive Care, San Martino Policlinico Hospital, Istituto di Ricovero e Cura a Carattere Scientifico (IRCCS) for Oncology and Neurosciences, Genoa, Italy; ^2^Department of Medicine, University of Barcelona, Barcelona, Spain; ^3^Griffith University School of Medicine, Gold Coast, QLD, Australia; ^4^Critical Care Research Group, The Prince Charles Hospital, Brisbane, QLD, Australia; ^5^Department of Anesthesiology and Perioperative Medicine, University of Utah, Salt Lake City, UT, United States; ^6^Faculty of Medicine, University of Queensland, Brisbane, QLD, Australia; ^7^Division of Neuroscience Critical Care, Departments of Neurology, Neurosurgery, and Anesthesiology and Critical Care Medicine, Johns Hopkins University School of Medicine, Baltimore, MD, United States; ^8^Hospital Sao Camilo de Esteio, Esteio, Brazil; ^9^Section of Cardiac Surgery, Department of Surgery, Max Rady College of Medicine, University of Manitoba, Winnipeg, MB, Canada; ^10^Cardiac Sciences Program, St. Boniface Hospital, Winnipeg, MB, Canada; ^11^Department of Surgery, Division of Cardiothoracic Surgery, Medical College of Wisconsin, Milwaukee, WI, United States; ^12^Division of Cardiovascular Diseases, Lankenau Medical Center and Lankenau Institute of Medical Research, Wynnewood, PA, United States; ^13^Jefferson Medical College, Philadelphia, PA, United States; ^14^Laboratório de Pneumologia LIM-09, Disciplina de Pneumologia, Heart Institute (Incor), Hospital das Clínicas da Faculdade de Medicina da Universidade de São Paulo, São Paulo, Brazil; ^15^Équipe de Recherche en Soins Intensifs (ERESI), Research Centre, Centre Intégré Universitaire de Santé et de Services Sociaux du Nord-de-l'île-de-Montréal, Hôpital du Sacré-Coeur-de-Montréal, 5400 boulevard Gouin Ouest, K-3000, Montreal, QC, Canada; ^16^Division of Critical Care Medicine, McGill University Health Centre, Montreal, QC, Canada; ^17^Department of Anesthesiology and Reanimation, Dr. Soetomo Academic Hospital, Surabaya, Indonesia; ^18^Institute for Clinical Research and Health Policy Studies, Tufts Medical Center/Tufts University School of Medicine, Boston, MA, United States; ^19^Pediatric Cardiac Intensive Care Division, National Cardiovascular Center Harapan Kita, Jakarta, Indonesia; ^20^Kuwait Extracorporeal Life Support Program, Ministry of Health, Kuwait City, Kuwait; ^21^Department of Anesthesia and Critical Care Medicine, Al-Amiri Hospital, Kuwait City, Kuwait; ^22^Department of Medicine 2, University Hospital, Ludwig Maximilian University of Munich, Munich, Germany; ^23^Department of Anesthesia and Critical Care, Fondazione IRCCS Ca' Granda, Ospedale Maggiore Policlinico, Milan, Italy; ^24^Emergency Department, Azienda Socio Sanitaria Territoriale (ASST) Monza - San Gerardo Hospital, Monza, Italy; ^25^University of Milano-Bicocca, Milan, Italy; ^26^Department of Surgical Sciences and Integrated Diagnostics, University of Genoa, Genoa, Italy; ^27^Department of Pathophysiology and Transplantation, Università degli Studi di Milano, Milan, Italy; ^28^Australian Centre for Health Services Innovation, Centre for Healthcare Transformation, School of Public Health and Social Work, Queensland University of Technology, Brisbane, QLD, Australia; ^29^Institut d'Investigacions Biomediques August Pi i Sunyer, Barcelona, Spain; ^30^Adult Intensive Care Services, The Prince Charles Hospital, Brisbane, QLD, Australia

**Keywords:** COVID-19, neurological complications, disability, stroke, neurological outcome

## Abstract

**Introduction:**

Neurological manifestations and complications in coronavirus disease-2019 (COVID-19) patients are frequent. Prior studies suggested a possible association between neurological complications and fatal outcome, as well as the existence of potential modifiable risk factors associated to their occurrence. Therefore, more information is needed regarding the incidence and type of neurological complications, risk factors, and associated outcomes in COVID-19.

**Methods:**

This is a pre-planned secondary analysis of the international multicenter observational study of the COVID-19 Critical Care Consortium (which collected data both retrospectively and prospectively from the beginning of COVID-19 pandemic) with the aim to describe neurological complications in critically ill COVID-19 patients and to assess the associated risk factors, and outcomes. Adult patients with confirmed COVID-19, admitted to Intensive Care Unit (ICU) will be considered for this analysis. Data collected in the COVID-19 Critical Care Consortium study includes patients' pre-admission characteristics, comorbidities, severity status, and type and severity of neurological complications. In-hospital mortality and neurological outcome were collected at discharge from ICU, and at 28-days.

**Ethics and Dissemination:**

The COVID-19 Critical Care Consortium main study and its amendments have been approved by the Regional Ethics Committee of participating sites. No further approval is required for this secondary analysis.

**Trial Registration Number:**

ACTRN12620000421932.

## Introduction

Coronavirus disease 2019 (COVID-19) presents with a wide spectrum of symptoms, from mild to severe, up to sequential organ failure and multiple-organ dysfunction (1). Reports of neurological manifestations associated with COVID-19 are increasing in the literature (2, 3). COVID-19 neurological signs can involve either the central nervous system (CNS), peripheral nervous system (PNS), or musculoskeletal system. Fatigue, myalgia, impaired sense of smell and taste, and headache are common neurological manifestations of COVID-19 (4, 5), whereas dizziness, confusion, delirium, agitation, stroke, hypoxic ischemic injury, seizures, encephalitis and coma among others have been reported neurological complications of hospitalized patients (4, 5). In some cases, neurological manifestations have been reported even without a primary respiratory involvement (4, 5). Several explanations have been proposed for the cause of neurological symptoms of COVID-19, but the underlying pathophysiology is not well defined. Putative mechanisms include viral neurotropism, a hyperinflammatory and hypercoagulable state, or pathological brain–lung crosstalk (6). Endothelial dysregulation (7–9) and pro-thrombotic state (10–12) have been widely suspected to be the possible main contributors of the increased risk of neurologic events. Indeed, COVID-19 patients are at high risk of hypoxia, hypotension, and microvascular abnormalities (13–15) which can promote neuroinflammation and excitotoxicity and increased permeability of the blood brain barrier (16). The risk is even more increased by the use of extracorporeal membrane oxygenation (ECMO) support that is a salvage option in COVID-19 critically ill patients with refractory hypoxemia (17). Prior studies suggested a possible association between neurological complications and mortality (18), but more information is required to delineate this association with respect to regional variation, as well as the risk factors associated to the occurrence of neurological complications (19). The aim of this study is to estimate the incidence of neurological complications in critically ill COVID-19 patients. Associations between neurological complications, patient-level variables and outcomes will also be assessed.

## Methods and Analysis

### Study Design

This is a pre-planned sub-analysis of a large international multicenter observational study of patients in participating intensive care units (ICUs) with COVID-19 of the COVID-19 Critical Care Consortium incorporating the ExtraCorporeal Membrane Oxygenation for 2019 novel Coronavirus Acute Respiratory Disease (ECMOCARD). The collaborative consists of investigators from the Asia-Pacific extracorporeal life support organization (APELSO) in collaboration with centers within the SPRINT-SARI and International Severe Acute Respiratory and emerging Infection Consortium (ISARIC) Network. In Australia, this study is also supported by collaboration with the “National registry on the treatment and outcomes of patients requiring ECMO” (EXCEL Registry). A panel of 13 experts in neurocritical care was created in 2020 together with the main protocol of the COVID-19 Critical Care Consortium by the Steering committee of the consortium. The panel planned this subanalysis and the electronic case report form (eCRF) in February 2020 and followed it up through monthly meeting. The study will be conducted in compliance with the STrengthening the Reporting of OBservational studies in Epidemiology (STROBE) (20) (Supplementary Item 1). Trial registration number: ACTRN12620000421932.

### Objectives

The primary objective is to identify and describe the type and incidence of neurological complications in COVID-19 patients before and after admission to ICU, for all ICU patients selected patient subgroups (sex, age, country, treatment, COVID-19 wave).

Secondary objectives include: To evaluate the effect of neurological complications on outcomes after COVID-19, i.e., mortality, duration of ICU and hospital stay, neurological outcome (modified Rankin scale, mRS) at discharge, incidence of delirium and cognitive outcome at discharge. To identify factors related to the occurrence of neurological complications (including neurological injury due to the antiviral therapy).

### Specific Sub-analysis

Secondary sub-analyses will also include the investigation of (1) magnetic resonance images (MRI) or computed tomography (CT) features; (2) serum biomarkers [neuronal injury markers (S100B, neuron specific enolase, NSE), endothelial dysfunction markers, inflammatory markers].

### Inclusion and Exclusion Criteria

The COVID-19 Critical Care Consortium included all COVID-19 patients (≥18 years) admitted to ICU for receiving critical care with confirmed or suspected COVID-19 respiratory disease. For this specific sub-analysis, further inclusion criteria will be available data on neurological complications/manifestations. Patients treated with mechanical ventilation or ECMO for other causes than COVID-19 will be excluded.

### Study Procedures and Setting

The protocol of the main study has been previously published (21). Participants in the COVID-19 Critical Care Consortium Observational Study are recruited at multiple sites in over 52 countries from 1st January 2020 onwards.

### Data Collection

**Figure 1:**
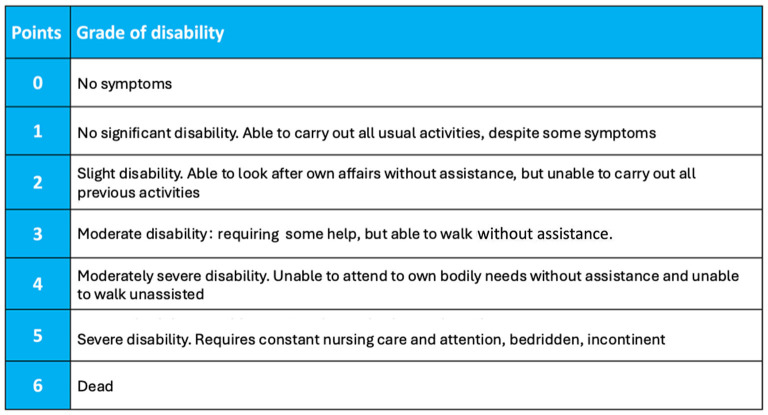
Modified Rankin Scale (mRS). The Modified Rankin Score (mRS) is a 6-point disability scale with possible scores ranging from 0 to 6 (from 0 = no symptoms to 6 = dead). A score of 0–3 indicate mild to moderate disability and a score of 4–5 indicate severe disability. From Wade (22).

Data collection started from the commencement of COVID-19 pandemic and is planned to continue until completion of COVID-19 pandemic, as judged by the World Health Organization. According to the COVID-19 Critical Care Consortium Observational Study protocol (21) and neurological sub-study protocol, the following data will be collected: general patient characteristics, age, gender, body mass index (BMI), country, previous chronic comorbidities, scores of severity; premorbid scores [modified Rankin scale (0–6 points), Figure 1; new neurological complications, laboratory findings, imaging, and management of neurological complications (Supplementary Item 2); patient outcome (mortality at discharge, at 28-days, withdrawal of life-saving therapy and reason; mRS at ICU discharge, mRS at 28 days after discharge). Main eCRF of the COVID-19 critical care consortium study and neuro sub-study are provided in the Supplementary Items 3, 4.

### Data Management

Data are stored in the central online eCRF database managed by the Oxford University in anonymized form, in order to preserve confidentiality of information in medical records. The Username and password will be assigned by the Oxford University during the registration process for individual Research Coordinators or Site Investigators. All electronic data transfer between study site and database will be username and password protected. The Participant List of the Neurology sub-study is maintained locally and is not to be transferred to any other location. confidentiality of the participant will be maintained unless disclosure is required by law.

Data entry and management will be coordinated by ISARIC and ECMOCARD steering committee, including programming and data management support. ANZIC-RC and ISARIC will act as custodian of the data. The University of Queensland (Australia) will receive data from the data custodians via data sharing agreements. The management committee of the trial will take responsibility for the content and integrity of any data.

### Definition of Neurological Complications

Definition of neurological complications (23–32) is listed in Table 1.

**Table 1:** Definition of neurological complications/manifestations.

**Neurological complication**	**Definition**
**Central nervous system**	
Ischemic stroke (23)	Neurological deficit due to an acute focal injury in the central nervous system caused by vascular involvement such as occlusion and cerebral infarction.
Intracranial hemorrhage (23, 24)	Bleeding that occurs inside the skull. Hemorrhagic stroke: neurological deficit due to an acute focal injury in the central nervous system caused by vascular involvement with intracerebral or subarachnoid hemorrhage. Subdural hematoma: collection of blood under the dura mater.
Encephalitis/meningitis (25)	Severe inflammatory disorder of the brain or meninges or parenchyma.
Transverse myelitis and other spinal cord pathologies (26)	Inflammatory disorder with acute or subacute motor-sensory and autonomic spinal cord dysfunction.
Epilepsy, seizures, and generalized convulsive status epilepticus (27, 28)	Epilepsy is a disorder of the brain characterized by an enduring predisposition to generate epileptic seizures, and by neurobiological, cognitive, psychological, and social consequences. Seizure is a transient occurrence of signs and/or symptoms due to abnormal excessive or synchronous neuronal activity in the brain. Generalized convulsive status epilepticus is defined in adults and children older than 5 years as ≥5 min of (1) continuous seizure or (2) two or more discrete seizures between which there is incomplete recovery of consciousness.
Delirium (29)	Acute change in consciousness and attention caused by an organic condition.
**Peripheral nervous system**	
Guillain-Barré Syndrome (30)	Inflammatory immune-mediated polyradiculoneuropathy with acute onset that manifests with tingling, progressive weakness, autonomic disfunction and pain.
Critical illness myopathy/neuropathy (31)	Neuromuscular weakness in the intensive care setting.
Hypogeusia/hyposmia (32)	Quantitative disorders characterized by reduction of taste or smell.
**Others**	
Hypoxic-ischemic brain injury (33)	Reduction in blood supply, oxygen supply or utilization that determines a decreased oxygen delivery to the brain and post cardiac arrest hypoxic ischemic brain injury (reduction in blood supply, oxygen supply or utilization that determines a decreased oxygen delivery to the brain due to cardiac arrest).

### Statistical Analysis Plan

Planned analyses will comprise of descriptive summaries and regression-based methods for estimating associations between patient-level variables, neurological complications, and outcomes. Descriptive statistics for summarizing the study cohort will be presented as medians with interquartile ranges and frequencies with percentages for continuous and categorical variables, respectively. As an observational study, missing data are expected; a data completeness summary will accompany descriptive summaries for all variables considered. The incidence of neurological complications will be calculated as the number of events per 1,000 ICU days and as the number of events divided by the total number of ICU admissions. Incidence will be estimated per complication using logistic and Poisson regression; Poisson models will include patient days as an offset to account for varying ICU exposure. Baseline models will be adjusted for patient-level variables (e.g., sex, age, country) and calendar time to account for the timing of different COVID-19 waves. Additional covariates will be informed by univariate analysis and penalized regression techniques to address the secondary objective related to incidence.

Analysis of associations between neurological complications and clinical outcomes will be examined using generalized linear mixed models for binary outcomes and parametric survival models for time-to-event outcomes. Evidence of potential associations, including patient demographics and clinical signs assessed during ICU admission, will initially be assessed using univariate analysis. Results of univariate analysis will be used to inform variable selection for multivariable analysis.

Multivariable models for all study objectives will be adjusted for known confounders as fixed or random effects, including study center, country, and calendar time. Model results will be presented as odds ratios (binary outcomes), relative risks (count outcomes) or hazard ratios (time-to-event outcomes) with 95% confidence intervals and *p*-values from hypothesis tests as appropriate.

### Study Status

The protocol version is 1.2.8 of the COVID-19 Critical Care Consortium Observational Study available at https://www.elso.org/COVID19/ECMOCARD.aspx. Data collection started from the commencement of COVID-19 pandemic and is planned to continue until completion of COVID-19 pandemic, as judged by the World Health Organization, as reported in the protocol.

## Discussion

This neurological sub-analysis of the COVID-19 Critical Care Consortium Observational Study is designed with the aim to obtain a detailed overview on neurological complications in a large international multicenter cohort of critically ill COVID-19 patients admitted to ICU, to determine incidence and risk factors of neurological complications, and the association of neurological complications with outcome. This study will provide real-time global data without geographic restrictions.

In the latest 2 years, knowledge has increased regarding extra-pulmonary complications of COVID-19 and their effect on outcome. Severe COVID-19 disease potentially involves multiple organs, including pulmonary, coagulation, cardiac, neurological, renal, hepatic, and gastrointestinal manifestations (34). Many neurological manifestations have been described recently in small observational studies, but additional evidence is needed from large multicentric cohorts. For this reason, in the present study we aim to depict the incidence, risk factors, and impact on outcome of neurological complications in critically ill COVID-19 patients from a large observational multicentric cohort. Data regarding pre-admission neurological manifestations, in-hospital neurological complications as well as ICU-and-hospital length of stay, neurological outcome (mRS), and mortality are available in the eCRF. This sub-analysis of the COVID-19 Critical Care Consortium Observational Study was pre-planned during the first/second wave of the pandemic (late 2020), thus increasing the data quality and minimizing the chance of spurious results and limiting the potential of exploratory learning. The number of patients included in the main study is continuously growing since the beginning of pandemic, allowing to obtain a large sample size, which can provide important information on the current incidence and characteristics of neurological manifestations in COVID-19 patients, evaluating potential associations between predictors and development of neurological complications, and assessing outcomes at discharge from ICU and from hospital and 28-days patients' outcomes. The included patients will be from different countries and centers, including low incoming countries. The patients will be also included during different waves and years of the pandemic, before and after the advent of vaccination campaigns, and with different variants of COVID-19 (i.e., omicron, delta, etc.). This will provide interesting insights on the differences in epidemiology, management strategies, geographical, and economical characteristics of COVID-19 adult patients who manifest neurological complications admitted to ICU. This global research context will provide the lens through which the study as well as its methodological approaches, findings, conclusions, and recommendations can be viewed.

### Incidence and Types of Neurological Manifestations and Complications of COVID-19

The importance of investigating neurological manifestations in COVID-19, assessing their risk factors, and association with outcome is justified by the increasing identification in the available literature of many studies which reported high morbidity and mortality and poor neurological outcome in COVID-19 patients who manifest neurological complications, with the need for identifying and investigating such alterations in a bigger cohort of COVID-19 critically ill patients. Indeed, regarding each of the identified neurological manifestations of COVID-19, the data are fragmentary and come from different small cohorts. Myalgia, dysgeusia, and taste dysfunction were frequently reported (33% of cases), altered mental status in 32%, headache 29%, encephalopathy 26%, alteration of consciousness 13%, stroke 12%, dizziness 10%, vision impairment 6%, intracerebral hemorrhage, 5%, seizure 4%, encephalitis 2%, and GBS 1% (35). Intracranial hemorrhage was identified in 477 patients with a prevalence of 0.85% and a mortality of 52% suggesting a very poor prognosis despite rare incidence (36). The prevalence of intracranial hemorrhage, ischemic stroke, and hypoxic ischemic brain injury was higher in patients with COVID-19 who underwent ECMO support (5.9%) with a mortality of 92% (17). Acute disseminated encephalomyelitis and acute hemorrhagic leukoencephalitis have been reported in 46 patients with COVID-19 only, of whom 32% died (37).

### Risk Factors for Neurological Manifestations and Complications of COVID-19

Regarding risk factors and association of neurological manifestations with outcome, a systematic review revealed that patients who suffer from a severe COVID-19 have more CNS involvement, neurological symptoms, and association with stroke. More severe patients had higher D-dimer and C-reactive protein levels than non-severe patients and presented multiple organ involvement (38). Myalgia, acute cerebrovascular disease, elevated creatin kinase, and lactate dehydrogenase were associated with more severe disease (3), while delirium on admission is a good predictor of mortality outcome in COVID-19 (39). In a cohort of 1,072 patients, age, headache at presentation, preexisting neurologic disease, invasive mechanical ventilation, and neutrophil/lymphocyte ratio ≥ 9 were independent predictors of new neurologic complications (40). In another study, the CT lung disease severity score was predictive of acute abnormalities on neuroimaging in patients with COVID-19 with neurologic manifestations (41). In a retrospective analysis, previous neurological history did not impact mortality, whereas new neurological manifestations were predictors of death (42). In a large cohort of 3,055 COVID-19 patients, preexisting neurological disorders were associated with higher risk of developing new neurological manifestations (2).

### Outcome of COVID-19 Patients With Neurological Manifestations and Complications

Patients affected by COVID-19 with neurological manifestations were noted to have an impaired quality of life in 49% of cases, with a residual disability at 6-months in 52%, impaired cognition in 69%, and persistence of anxiety and depression in 32% (43). Neurological outcome in 135 patients with COVID-19 at 3-months follow-up was impaired (44), and a significant patient number still suffer from neurological sequelae 1 year after SARS-CoV-2 infection (45). A large multicentric study investigating delirium in 4,530 COVID-19 patients revealed that acute brain dysfunction was highly prevalent and prolonged in critically ill patients with COVID-19, with benzodiazepines and lack of family visitation identified to be risk factors for its development (46). After 6 months, in a cohort of 236,379 patients with COVID-19, neurological and psychiatric manifestations had an estimated incidence of 33.62 and 12.84%, respectively (47). Clinical outcome was evaluated in a cohort of 267 patients, concluding that patients with cerebrovascular disease had the worst prognosis (48).

### Potential Pitfalls and Unintended Effects of This Study

Taken together, a large number of case reports and case series, despite coming mainly from small cohorts and local studies raise interest around the need for clarification about type and incidence of COVID-19 neurological manifestations, risk factors, and association with outcome on large scale, thus encouraging to better plan for possible management and therapeutics for neurological complications in critically ill COVID-19 patients. A limitation of current available data in the literature is that most of the data come from small cohorts, that could be addressed by using the larger COVID-19 Critical Care Consortium. Our study is unique in a way that we can address both limitations by studying the questions with international cohort with granular neurological variables. According to the design of our study, no unintended effects are expected. However, some limitations should be addressed. Being an observational study, it can be exposed to bias and confounding. Additionally, it cannot be used to demonstrate causality.

## Conclusions

In conclusion the present study will provide new information on a global scale regarding the incidence and type of neurological complications, risk factors, and associated outcomes in COVID-19 with clinical applications.

## Abbreviations

APELSO: Asia-Pacific extracorporeal life support organization
BMI: body mass index
CNS: central nervous system
COVID-19: Coronavirus disease 2019
CT: computed tomography
ECMO: extracorporeal membrane oxygenation
ECMOCARD: ExtraCorporeal Membrane Oxygenation for 2019 novel Coronavirus Acute Respiratory Disease
eCRF: electronic case report form
ICU: intensive care unit
IRB: institutional review board
ISARIC: International Severe Acute Respiratory and emerging Infection Consortium
MRI: magnetic resonance images
mRS: modified Rankin scale
NSE: neuron specific enolase
PNS: peripheral nervous system.

## Appendix

**Table:**

**Prefix/First Name/Last Name**	**Site Name**
Eugeni Roure Marta Roure	The University of Queensland, Australia
Fatima Nasrallah	The Queensland Brain Institute, The University of Queensland, St. Lucia, QLD, Australia
Katie McMahon	School of Clinical Sciences and Centre for Biomedical Technologies, Queensland University of Technology, Brisbane, Queensland, Australia
Judith Bellapart	Royal Brisbane and Women's Hospital, Herston, Queensland, Australia.
Fabio Silvio Taccone	Erasmus Hospital, Free University of Brussels, Evere, Belgium
Tala Al-Dabbous Huda Alfoudri Mohammed Shamsah	Al Adan Hospital
Subbarao Elapavaluru Ashley Berg Christina Horn	Allegheny General Hospital
Yunis Mayasi	Avera McKennan Hospital & University Health Centre
Stephan Schroll	Barmherzige Bruder Regansburg
Dan Meyer Jorge Velazco Ludmyla Ploskanych Wanda Fikes Rohini Bagewadi Marvin Dao Haley White Alondra Berrios Laviena Ashley Ehlers Maysoon Shalabi-McGuire Trent Witt	Baylor Scott & White Health
Lorenzo Grazioli Luca Lorini	Bergamo Hospital
E. Wilson Grandin Jose Nunez Tiago Reyes	Beth Israel Deaconess Medical Centre
Diarmuid O'Briain Stephanie Hunter	Box Hill Hospital
Mahesh Ramanan Julia Affleck	Caboolture Hospital
Hemanth Hurkadli Veerendra Sumeet Rai Josie Russell-Brown Mary Nourse	Canberra Hospital
Mark Joseph Brook Mitchell Martha Tenzer	Carilion Clinic
Ryuzo Abe	Chiba University Graduate School of Medicine
Hwa Jin Cho In Seok Jeong	Chonnam National University Hospital
Nadeem Rahman Vivek Kakar	Cleveland Clinic- Abu Dhabi
Nicolas Brozzi	Cleveland Clinic - Florida
Omar Mehkri Sudhir Krishnan Abhijit Duggal Stuart Houltham	Cleveland Clinic - Ohio
Jerónimo Graf	Clinica Alemana De Santiago
Roderigo Diaz Roderigo Orrego Camila Delgado Joyce González Maria Soledad Sanchez Michael Piagnerelli Josefa Valenzuela Sarrazin	Clinica Las Condez
A/Prof. Gustavo Zabert Lucio Espinosa Paulo Delgado Victoria Delgado	Clinica Pasteur National- University of Comahue
Diego Fernando Bautista Rincón Angela Maria Marulanda Yanten Melissa Bustamante Duque	Clinica Valle de Lilli
Daniel Brodie	Medical ICU, Columbia College of Physicians and Surgeons, New-York-Presbyterian Hospital, NY, NY, USA
Alyaa Elhazmi Abdullah Al-Hudaib	Dr Sulaiman Alhabib Medical Group – Research Center, Riyadh, Saudi Arabia
Maria Callahan	Emory University Healthcare System
M. Azhari Taufik Elizabeth Yasmin Wardoyo Margaretha Gunawan Nurindah S Trisnaningrum Vera Irawany Muhammad Rayhan	Fatmawati Hospital
Mauro Panigada Antonio Pesenti Alberto Zanella Giacomo Grasselli Sebastiano Colombo Chiara Martinet Gaetano Florio	Fondazione IRCCS Policlinico of Milan (Fondazione IRCCS Ca' Granda Ospedale Maggiore Policlinico)
Massimo Antonelli Simone Carelli Domenico L. Grieco	Fondazione Policlinico Universitario Agostino Gemelli IRCCS
Motohiro Asaki	Fujieda Municipal General Hospital
Kota Hoshino	Fukuoka University
Leonardo Salazar Mary Alejandra Mendoza Monsalve	Fundación Cardiovascular de Colombia
John Laffey Bairbre McNicholas David Cosgrave	Galway University Hospitals
Joseph McCaffrey Allison Bone	Geelong Hospital
Yusuff Hakeem	Glenfield Hospital
James Winearls Mandy Tallott	Gold Coast University Hospital
David Thomson Christel Arnold-Day Jerome Cupido Zainap Fanie Malcom Miller Lisa Seymore Dawid van Straaten	Groote Schuur Hospital
Ali Ait Hssain Jeffrey Aliudin Al-Reem Alqahtani Khoulod Mohamed Ahmed Mohamed Darwin Tan Joy Villanueva Ahmed Zaqout	Hamad General Hospital - Weill Cornell Medical College in Qatar
Ethan Kurtzman Arben Ademi	
Ana Dobrita Khadija El Aoudi Juliet Segura	Hartford HealthCare
Gezy Giwangkancana	Hasan Sadikin Hospital (Adult)
Shinichiro Ohshimo	Hiroshima University
Javier Osatnik	Hospital Alemán
Anne Joosten	Hospital Civil Marie Curie
Antoni Torres Minlan Yang Ana Motos	Hospital Clinic, Barcelona
Carlos Luna	Hospital de Clínicas
Francisco Arancibia	Hospital del Tórax
Virginie Williams Alexandre Noel	Hospital du Sacre Coeur (Universite de Montreal)
Nestor Luque	Hospital Emergencia Ate Vitarte
Marina Fantini	Hospital Mater Dei
Ruth Noemi Jorge García Enrique Chicote Alvarez	Hospital Nuestra Señora de Gracia
Anna Greti	Hospital Puerta de Hierro
Adrian Ceccato	Hospital Universitari Sagrat Cor
Angel Sanchez	Hospital Universitario Sant Joan d'Alacant
Ana Loza Vazquez	Hospital Universitario Virgen de Valme
Ferran Roche-Campo Diego Franch-Llasat	Hospital Verge de la Cinta de Tortosa
Divina Tuazon	Houston Methodist Hospital
Marcelo Amato Luciana Cassimiro Flavio Pola Francis Ribeiro Guilherme Fonseca	INCOR (Universidade de São Paulo)
Heidi Dalton Mehul Desai Erik Osborn Hala Deeb	INOVA Fairfax Hospital
Antonio Arcadipane Gennaro Martucci Giovanna Panarello Chiara Vitiello Claudia Bianco Giovanna Occhipinti Matteo Rossetti Raffaele Cuffaro	ISMETT
Sung-Min Cho Glenn Whitman	Johns Hopkins
Hiroaki Shimizu Naoki Moriyama	Kakogawa Acute Care Medical Center
Jae-Burm Kim	Keimyung University Dong San Hospital
Nobuya Kitamura	Kimitsu Chuo Hospital
Johannes Gebauer	Klinikum Passau
Toshiki Yokoyama	Kouritu Tousei Hospital
Abdulrahman Al-Fares Sarah Buabbas Esam Alamad Fatma Alawadhi	
Kalthoum Alawadi	Al-Amiri and Jaber Al-Ahmed Hospitals, Kuwait Extracorporeal Life Support Program
Hiro Tanaka	Kyoto Medical Centre
Satoru Hashimoto Masaki Yamazaki	Kyoto Prefectural University of Medicine
Tak-Hyuck Oh	Kyung Pook National University Chilgok Hospital
Mark Epler Cathleen Forney	
Louise Kruse Jared Feister Joelle Williamson Katherine Grobengieser	Lancaster General Health
Eric Gnall Sasha Golden Mara Caroline Timothy Shapiro Colleen Karaj Lisa Thome Lynn Sher Mark Vanderland Mary Welch Sherry McDermott	Lankenau Institute of Medical Research (Main Line Health)
Matthew Brain Sarah Mineall	Launceston General Hospital
Dai Kimura	Le Bonheur Children's Hospital
Luca Brazzi Gabriele Sales Giorgia Montrucchio	Le Molinette Hospital (Ospedale Molinette Torino)
Tawnya Ogston	Legacy Emanuel Medical Center
Dave Nagpal Karlee Fischer	London Health Sciences Centre
Roberto Lorusso	Maastricht University Medical Centre
Rajavardhan Rangappa Sujin Rai Argin Appu	Manipal Hospital Whitefield
Mariano Esperatti Nora Angélica Fuentes Maria Eugenia Gonzalez	Hospital Privado de Comunidad. Mar del Plata. Escuela Superior de Medicina. Universidad Nacional de Mar del Plata
Diarmuid O'Briain	Maroondah Hospital
Edmund G. Carton	Mater Misericordiae University Hospital
Ayan Sen Amanda Palacios Deborah Rainey	Mayo Clinic College of Medicine
Gordan Samoukoviv Josie Campisi	McGill University Health Centre
Lucia Durham Emily Neumann Cassandra Seefeldt Octavio Falcucci Amanda Emmrich Jennifer Guy Carling Johns Kelly Potzner Catherine Zimmermann Angelia Espinal	Medical College of Wisconsin (Froedtert Hospital)
Nina Buchtele Michael Schwameis Andrea Korhnfehl Roman Brock Thomas Staudinger	Medical University of Vienna
Stephanie-Susanne Stecher Michaela Barnikel Sófia Antón Alexandra Pawlikowski	Medical Department II, LMU Hospital Munich
Akram Zaaqoq Lan Anh Galloway Caitlin Merley	MedStar Washington Hospital Centre
Alistair Nichol	Monash University
Marc Csete Luisa Quesada Isabela Saba	Mount Sinai Medical Centre
Daisuke Kasugai Hiroaki Hiraiwa	
Taku Tanaka	Nagoya University Hospital
Eva Marwali Yoel Purnama Santi Rahayu Dewayanti Ardiyan Dafsah Arifa Juzar Debby Siagian	National Cardiovascular Center Harapan Kita, Jakarta, Indonesia
Yih-Sharng Chen	National Taiwan University Hospital
Mark Ogino	Nemours Alfred I duPont Hospital for Children
Indrek Ratsep Andra-Maris Post Piret Sillaots Anneli Krund Merili-Helen Lehiste Tanel Lepik	North Estonia Medical Centre
Frank Manetta Effe Mihelis Iam Claire Sarmiento Mangala Narasimhan Michael Varrone	Northwell Health
Mamoru Komats	Obihiro-Kosei General Hospital
Julia Garcia-Diaz Catherine Harmon	Ochsner Clinic Foundation
S. Veena Satyapriya Amar Bhatt Nahush A. Mokadam Alberto Uribe Alicia Gonzalez Haixia Shi Johnny McKeown Joshua Pasek Juan Fiorda Marco Echeverria	Ohio State University Medical Centre
Rita Moreno	Oklahoma Heart Institute
Bishoy Zakhary	Oregon Health and Science University Hospital (OHSU)
Marco Cavana Alberto Cucino	Ospedale di Arco (Trento Hospital)
Giuseppe Foti Marco Giani Benedetta Fumagalli	Ospedale San Gerardo
Davide Chiumello Valentina Castagna	Ospedale San Paolo
Andrea Dell'Amore Paolo Navalesi	Padua University Hospital (Policlinico of Padova)
Hoi-Ping Shum	Pamela Youde Nethersole Eastern Hospital
Alain Vuysteke	Papworth Hospitals NHS Foundation Trust
Asad Usman Andrew Acker Benjamin Smood Blake Mergler Federico Sertic Madhu Subramanian Alexandra Sperry Nicolas Rizer	Penn Medicine (Hospital of the University of Pennsylvania)
Erlina Burhan Menaldi Rasmin Ernita Akmal Faya Sitompul Navy Lolong Bhat Naivedh	Persahabatan General Hospital
Simon Erickson	Perth Children's Hospital
Peter Barrett David Dean	
Julia Daugherty	Piedmont Atlanta Hospital
Antonio Loforte	Policlinico di S. Orsola, Università di Bologna
Irfan Khan Mohammed Abraar Quraishi Olivia DeSantis	Presbyterian Hospital Services, Albuquerque
Dominic So Darshana Kandamby	Princess Margaret Hospital
Jose M. Mandei Hans Natanael	Prof Dr R. D. Kandou General Hospital - Paediatric
Eka YudhaLantang Anastasia Lantang	Prof Dr R. D R. D. Kandou General Hospital - Adult
Surya Oto Wijaya	Dr Sulianti Saroso Hospital
Anna Jung	Providence Saint John's Health Centre
George Ng Wing Yiu Ng	Queen Elizabeth Hospital, Hong Kong
Pauline Yeung Ng Shu Fang	The University of Hong Kong
Alexis Tabah Megan Ratcliffe Maree Duroux	Redcliffe Hospital
Shingo Adachi Shota Nakao	Rinku General Medical Center (and Senshu Trauma and Critical Care Center)
Pablo Blanco Ana Prieto Jesús Sánchez	Rio Hortega University Hospital
Meghan Nicholson	Rochester General Hospital
Warwick Butt Alyssa Serratore Carmel Delzoppo	Royal Children's Hospital
Pierre Janin Elizabeth Yarad	Royal North Shore Hospital
Richard Totaro Jennifer Coles	Royal Prince Alfred Hospital
Bambang Pujo	RSUD Soetomo
Robert Balk Andy Vissing Esha Kapania James Hays Samuel Fox	
Garrett Yantosh Pavel Mishin	Rush University, Chicago
Saptadi Yuliarto Kohar Hari Santoso Susanthy Djajalaksana	Saiful Anwar Malang Hospital (Brawijaya University) (Paediatrics)
Arie Zainul Fatoni	Saiful Anwar Malang Hospital (Brawijaya University) (Adult)
Masahiro Fukuda	Saiseikai Senri Hospital
Keibun Liu	Saiseikai Utsunomiya Hospital
Paolo Pelosi Denise Battaglini	San Martino Hospital
Juan Fernando Masa Jiménez	San Pedro de Alcantara Hospital
Diego Bastos	Sao Camilo Cura D'ars
Sérgio Gaião	São João Hospital Centre, Porto
Desy Rusmawatiningtyas	Sardjito Hospital (Paediatrics)
Young-Jae Cho	Seoul National University Bundang Hospital
Su Hwan Lee	Severance Hospital
Tatsuya Kawasaki	Shizuoka Children's Hospital
Laveena Munshi	Sinai Health Systems (Mount Sinai Hospital)
Pranya Sakiyalak Prompak Nitayavardhana	Siriraj Hospital
Tamara Seitz	Sozialmedizinisches Zentrum Süd – Kaiser-Franz-Josef-Spital
Rakesh Arora David Kent	St Boniface Hospital (University of Mannitoba)
Daniel Marino	St Christopher's Hospital for Children
Swapnil Parwar Andrew Cheng Jennene Miller	St George Hospital
Shigeki Fujitani Naoki Shimizu	St Marianna Medical University Hospital
Jai Madhok Clark Owyang	Stanford University Hospital
Hergen Buscher Claire Reynolds	St Vincent's Hospital
Olavi Maasikas AleksanBeljantsev Vladislav Mihnovits	Tartu University Hospital
Takako Akimoto Mariko Aizawa Kanako Horibe Ryota Onodera	Teine Keijinkai Hospital
Carol Hodgson Aidan Burrell Meredith Young	The Alfred Hospital
Timothy George	The Heart Hospital Baylor Plano, Plano
Kiran Shekar Niki McGuinness Lacey Irvine	The Prince Charles Hospital
Brigid Flynn	The University of Kansas Medical Centre
Tomoyuki Endo	Tohoku Medical and Pharmaceutical University
Kazuhiro Sugiyama	Tokyo Metropolitan Bokutoh Hospital
Keiki Shimizu	Tokyo Metropolitan Medical Center
Eddy Fan Kathleen Exconde	Toronto General Hospital
Shingo Ichiba	Tokyo Women's Medical University Hospital
Leslie Lussier	Tufts Medical Centre (and Floating Hospital for Children)
Gösta Lotz	Universitätsklinikum Frankfurt (University Hospital Frankfurt) (Uniklinik)
Maximilian Malfertheiner Lars Maier Esther Dreier	Universitätsklinikum Regensburg (Klinik für Innere Medizin II)
Neurinda Permata Kusumastuti	University Airlangga Hospital (Paediatric)
Colin McCloskey Al-Awwab Dabaliz Tarek B Elshazly Josiah Smith	University Hospital Cleveland Medical Centre (UH Cleveland Hospital)
Konstanty S. Szuldrzynski Piotr Bielański	University Hospital in Krakow
Yusuff Hakeem	University Hospitals of Leicester NHS Trust (Glenfield Hospital)
Keith Wille	University of Alabama at Birmingham Hospital (UAB)
Srinivas Murthy	University of British Columbia
Ken Kuljit S. Parhar Kirsten M. Fiest Cassidy Codan Anmol Shahid	University of Calgary (Peter Lougheed Centre, Foothills Medical Centre, South Health Campus and Rockyview General Hospital)
Mohamed Fayed Timothy Evans Rebekah Garcia Ashley Gutierrez Hiroaki Shimizu	University of California, San Francisco-Fresno Clinical Research Centre
Tae Song Rebecca Rose	University of Chicago
Suzanne Bennett Denise Richardson	University of Cincinnati Medical Centre
Giles Peek	University of Florida
Lovkesh Arora Kristina Rappapport Kristina Rudolph Zita Sibenaller Lori Stout Alicia Walter	University of Iowa
Daniel Herr Nazli Vedadi	University of Maryland - Baltimore
Robert Bartlett	University of Michigan Medical Center
Antonio Pesenti	University of Milan
Shaun Thompson Julie Hoffman Xiaonan Ying	University of Nebraska Medical Centre
Ryan Kennedy	University of Oklahoma Health Sciences Centre (OU)
Muhammed Elhadi	Faculty of Medicine, University of Tripoli
Matthew Griffee Anna Ciullo Yuri Kida	University of Utah Hospital
Ricard Ferrer Roca JordI Riera Sofia Contreras Cynthia Alegre	Vall d'Hebron University Hospital, Barcelona
Christy Kay Irene Fischer Elizabeth Renner	Washington University in St. Louis/ Barnes Jewish Hospital
Hayato Taniguci	Yokohama City University Medical Center
John Fraser Gianluigi Li Bassi Jacky Suen	
Adrian Barnett Nicole White Kristen Gibbons Simon Forsyth Amanda Corley India Pearse Samuel Hinton Gabriella Abbate Halah Hassan Silver Heinsar Varun A Karnik Katrina Ki Hollier F. O'Neill Nchafatso Obonyo Leticia Pretti Pimenta Janice D. Reid Kei Sato Kiran Shekar Aapeli Vuorinen Karin S. Wildi Emily S. Wilson Stephanie Yerkovich	COVID-19 Critical Care Consortium
James Lee Daniel Plotkin Barbara Wanjiru Citarella Laura Merson	ISARIC, Centre for Tropical Medicine and Global Health, University of Oxford, Oxford, UK
