# Correction: Neurological manifestations of SARS-CoV-2 infection: protocol for a sub-analysis of the COVID-19 critical care consortium observational study

**DOI:** 10.3389/fmed.2026.1848413

**Published:** 2026-05-01

**Authors:** Denise Battaglini, Lavienraj Premraj, Matthew Griffee, Samuel Huth, Jonathon Fanning, Glenn Whitman, Diego Bastos Porto, Rakesh Arora, Lucian Durham, Eric Gnall, Marcelo Amato, Virginie Williams, Alexandre Noel, Sabrina Araujo De Franca, Gordan Samoukovic, Bambang Pujo, David Kent, Eva Marwali, Abdulrahman Al-Fares, Stephanie-Susanne Stecher, Mauro Panigada, Marco Giani, Giuseppe Foti, Paolo Pelosi, Antonio Pesenti, Nicole Marie White, Gianluig Li Bassi, Jacky Suen, John F. Fraser, Chiara Robba, Sung-Min Cho

**Affiliations:** ^1^Anesthesia and Intensive Care, San Martino Policlinico Hospital, Istituto di Ricovero e Cura a Carattere Scientifico (IRCCS) for Oncology and Neurosciences, Genoa, Italy; ^2^Department of Medicine, University of Barcelona, Barcelona, Spain; ^3^Griffith University School of Medicine, Gold Coast, QLD, Australia; ^4^Critical Care Research Group, The Prince Charles Hospital, Brisbane, QLD, Australia; ^5^Department of Anesthesiology and Perioperative Medicine, University of Utah, Salt Lake City, UT, United States; ^6^Faculty of Medicine, University of Queensland, Brisbane, QLD, Australia; ^7^Division of Neuroscience Critical Care, Departments of Neurology, Neurosurgery, and Anesthesiology and Critical Care Medicine, Johns Hopkins University School of Medicine, Baltimore, MD, United States; ^8^Hospital Sao Camilo de Esteio, Esteio, Brazil; ^9^Section of Cardiac Surgery, Department of Surgery, Max Rady College of Medicine, University of Manitoba, Winnipeg, MB, Canada; ^10^Cardiac Sciences Program, St. Boniface Hospital, Winnipeg, MB, Canada; ^11^Department of Surgery, Division of Cardiothoracic Surgery, Medical College of Wisconsin, Milwaukee, WI, United States; ^12^Division of Cardiovascular Diseases, Lankenau Medical Center and Lankenau Institute of Medical Research, Wynnewood, PA, United States; ^13^Jefferson Medical College, Philadelphia, PA, United States; ^14^Laboratório de Pneumologia LIM-09, Disciplina de Pneumologia, Heart Institute (Incor), Hospital das Clínicas da Faculdade de Medicina da Universidade de São Paulo, São Paulo, Brazil; ^15^Équipe de Recherche en Soins Intensifs (ERESI), Research Centre, Centre Intégré Universitaire de Santé et de Services Sociaux du Nord-de-l'île-de-Montréal, Hôpital du Sacré-Coeur-de-Montréal, 5400 boulevard Gouin Ouest, K-3000, Montreal, QC, Canada; ^16^Division of Critical Care Medicine, McGill University Health Centre, Montreal, QC, Canada; ^17^Department of Anesthesiology and Reanimation, Dr. Soetomo Academic Hospital, Surabaya, Indonesia; ^18^Institute for Clinical Research and Health Policy Studies, Tufts Medical Center/Tufts University School of Medicine, Boston, MA, United States; ^19^Pediatric Cardiac Intensive Care Division, National Cardiovascular Center Harapan Kita, Jakarta, Indonesia; ^20^Kuwait Extracorporeal Life Support Program, Ministry of Health, Kuwait City, Kuwait; ^21^Department of Anesthesia and Critical Care Medicine, Al-Amiri Hospital, Kuwait City, Kuwait; ^22^Department of Medicine 2, University Hospital, Ludwig Maximilian University of Munich, Munich, Germany; ^23^Department of Anesthesia and Critical Care, Fondazione IRCCS Ca' Granda, Ospedale Maggiore Policlinico, Milan, Italy; ^24^Emergency Department, Azienda Socio Sanitaria Territoriale (ASST) Monza - San Gerardo Hospital, Monza, Italy; ^25^University of Milano-Bicocca, Milan, Italy; ^26^Department of Surgical Sciences and Integrated Diagnostics, University of Genoa, Genoa, Italy; ^27^Department of Pathophysiology and Transplantation, Università degli Studi di Milano, Milan, Italy; ^28^Australian Centre for Health Services Innovation, Centre for Healthcare Transformation, School of Public Health and Social Work, Queensland University of Technology, Brisbane, QLD, Australia; ^29^Institut d'Investigacions Biomediques August Pi i Sunyer, Barcelona, Spain; ^30^Adult Intensive Care Services, The Prince Charles Hospital, Brisbane, QLD, Australia

**Keywords:** COVID-19, neurological complications, disability, stroke, neurological outcome

An error was identified in Figure 1 of the originally published article, specifically concerning the classification of the modified Rankin Scale (mRS) domains. The figure has been corrected and replaced accordingly. The revised version of Figure 1 is provided below.

**Figure 1:**
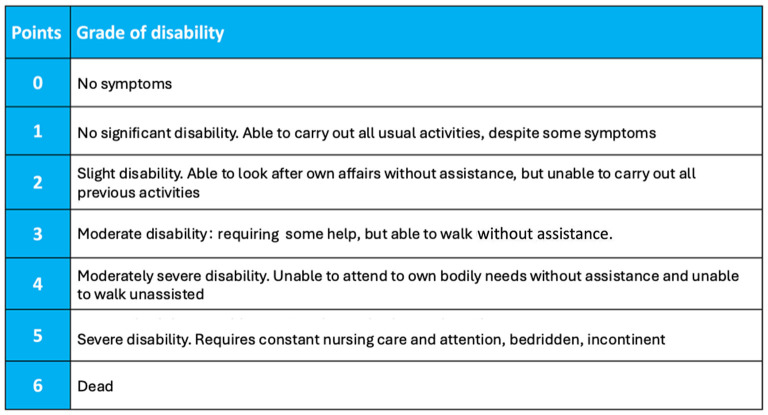
Modified Rankin Scale (mRS). The Modified Rankin Score (mRS) is a 6-point disability scale with possible scores ranging from 0 to 6 (from 0 = no symptoms to 6 = dead). A score of 0–3 indicate mild to moderate disability and a score of 4–5 indicate severe disability. From Wade (22).

The original version of this article has been updated.

