# Supplementary material for: Neurological Manifestations of SARS-CoV-2 Infection: Protocol for a Sub-analysis of the COVID-19 Critical Care Consortium Observational Study
Source: Front Med (Lausanne). 2022 Jul 22;9:930217. doi: 10.3389/fmed.2022.930217 (PMC9355612; doi:10.3389/fmed.2022.930217)
Supplement: Supplementary file 1 [file Data_Sheet_1.DOCX]

Supplementary Material

**Neurological manifestations of SARS-CoV-2 infection: protocol for a sub-analysis of the COVID-19 Critical Care Consortium Observational Study**

Table of content:

Item 1. STROBE checklist. [page 2-3]

Item 2. Data collection on new neurological complications. [page 4]

Item 3. Assessment schedule of the COVID-19 critical care consortium main study. [page 5]

Item 4. Assessment schedule of the COVID-19 critical care consortium neurological sub-study. [page 6-7]

**Item 1.** STROBE Statement—checklist of items that should be included in reports of observational studies

|  | Item No | Recommendation | Page  No |
| --- | --- | --- | --- |
| **Title and abstract** | 1 | (*a*) Indicate the study’s design with a commonly used term in the title or the abstract | 2-3 |
|  |  | (*b*) Provide in the abstract an informative and balanced summary of what was done and what was found | 2-3 |
| Introduction | | | |
| Background/rationale | 2 | Explain the scientific background and rationale for the investigation being reported | 4-5 |
| Objectives | 3 | State specific objectives, including any prespecified hypotheses | 4-5 |
| Methods | | | |
| Study design | 4 | Present key elements of study design early in the paper | 5-7 |
| Setting | 5 | Describe the setting, locations, and relevant dates, including periods of recruitment, exposure, follow-up, and data collection | 5-7 |
| Participants | 6 | (*a*) *Cohort study*—Give the eligibility criteria, and the sources and methods of selection of participants. Describe methods of follow-up  *Case-control study*—Give the eligibility criteria, and the sources and methods of case ascertainment and control selection. Give the rationale for the choice of cases and controls  *Cross-sectional study*—Give the eligibility criteria, and the sources and methods of selection of participants | 5-7 |
|  |  | (*b*) *Cohort study*—For matched studies, give matching criteria and number of exposed and unexposed  *Case-control study*—For matched studies, give matching criteria and the number of controls per case | 5-7 |
| Variables | 7 | Clearly define all outcomes, exposures, predictors, potential confounders, and effect modifiers. Give diagnostic criteria, if applicable | 5-7 |
| Data sources/ measurement | 8* | For each variable of interest, give sources of data and details of methods of assessment (measurement). Describe comparability of assessment methods if there is more than one group | *5-10* |
| Bias | 9 | Describe any efforts to address potential sources of bias | 5-7 |
| Study size | 10 | Explain how the study size was arrived at |  |
| Quantitative variables | 11 | Explain how quantitative variables were handled in the analyses. If applicable, describe which groupings were chosen and why | 9-10 |
| Statistical methods | 12 | (*a*) Describe all statistical methods, including those used to control for confounding | 9-10 |
|  |  | (*b*) Describe any methods used to examine subgroups and interactions | 9-10 |
|  |  | (*c*) Explain how missing data were addressed | 9-10 |
|  |  | (*d*) *Cohort study*—If applicable, explain how loss to follow-up was addressed  *Case-control study*—If applicable, explain how matching of cases and controls was addressed  *Cross-sectional study*—If applicable, describe analytical methods taking account of sampling strategy | 9-10 |
|  |  | (*e*) Describe any sensitivity analyses | 9-10 |

**Item 2. Data collection on new neurological complications**

New neurological complications [diagnosis of ischemic stroke; intracranial hemorrhage; hypoxic ischemic brain injury, meningitis/ encephalitis; transverse myelitis/ spinal cord pathology; occurrence of seizure; delirium; other CNS complications; Guillain Barré syndrome (GBS); critical illness myopathy (CIM)/ neuropathy (CIN)]; diagnostic scores [Toast stroke classification; National Institutes of Health Stroke Score, intracranial hemorrhage classification and score, classification of spinal cord pathology]; brain imaging type and findings [computed tomography (CT), magnetic resonance image (MRI), other]; management of ischemic stroke [fibrinolysis, endovascular thrombectomy, antiplatelet agent, anticoagulation, hemicraniectomy]; ancillary testing for hypoxic ischemic brain injury [CT, MRI, electroencephalogram (EEG), somatosensory evoked potentials, biochemistry, other]; ancillary testing for meningitis/ encephalitis [CT, MRI, CSF study, EEG, others]; simultaneous drug use for seizure management [benzodiazepines, barbiturate, phenytoin, valproate, levetiracetam, other]; investigations for GBS [electromyography (EMG), CSF study, biochemistry, other]; treatment of GBS [IV-Ig, plasma exchange, other]; investigations for CIM/ CIN [EMG, CSF study, biochemistry, other]; treatment of CIM/ CIN [IV-Ig, plasma exchange, other]; olfactory and taste changes; serum biochemistry [biomarkers (S100b, NSE)].

**Item 3. Assessment schedule of the COVID-19 critical care consortium main study.**

Table legend: ABG, blood gas analysis; ECMO, extracorporeal membrane oxygenation; MV, mechanical ventilation.

|  | **Screening** | **ICU admission** | **Start MV** | **Start ECMO** | **Daily** | **Outcomes** |
| --- | --- | --- | --- | --- | --- | --- |
| Eligibility criteria | x |  |  |  |  |  |
| Demographics |  | x |  |  |  |  |
| Comorbidities |  | x |  |  |  |  |
| Severity scoring |  | x |  |  |  |  |
| Symptoms |  | x |  |  |  |  |
| ABG and biochemistry |  | x | x | x | x |  |
| Respiratory support |  |  | x | x | x |  |
| Adjunctive therapies |  |  | x | x | x |  |
| ECMO parameters |  |  |  | x | x |  |
| Pulmonary mechanics |  |  |  | x | x |  |
| Microbiology |  |  |  |  | x |  |
| Blood transfusion |  |  |  |  | x |  |
| Length of stay |  |  |  |  |  | x |
| Survival |  |  |  |  |  | x |
|  | | | | | | |

**Item 4. Assessment schedule of the COVID-19 critical care consortium neurological sub-study.**

|  | **Screening** | **ICU admission** | **Day of**  **Complication/ assessment** | **Outcomes** |  |
| --- | --- | --- | --- | --- | --- |
| Eligibility criteria | x |  |  |  |  |
| Demographics |  | x |  |  |  |
| Neurologic comorbidities |  | x |  |  |  |
| Pre-morbid mRS |  | x |  |  |  |
| Ischemic stroke (yes/no) |  |  | x |  |  |
| Stroke TOAST classification |  |  | x |  |  |
| Stroke, NIHSS |  |  | x |  |  |
| Imaging of stroke |  |  | x |  |  |
| Management therapy of stroke |  |  | x |  |  |
| Intracranial hemorrhage (yes/no) |  |  | x |  |  |
| ICH classification |  |  | x |  |  |
| Imaging of ICH |  |  | x |  |  |
| Management therapy of ICH |  |  | x |  |  |
| Hypoxic-ischemic brain injury (yes/no) |  |  | x |  |  |
| Ancillary testing |  |  | x |  |  |
| Meningitis/ encephalitis (yes/no) |  |  | x |  |  |
| Diagnostic testing |  |  | x |  |  |
| Spinal cord injury (yes/no) |  |  | x |  |  |
| Diagnostic testing |  |  | x |  |  |
| Seizures (yes/no) |  |  | x |  |  |
| Drugs |  |  | x |  |  |
| N. days with seizures |  |  | x | x |  |
| Delirium (yes/no) |  |  | x |  |  |
| Delirium classification |  |  | x |  |  |
| Other CNS involvement (yes/no) |  |  | x |  |  |
| Type |  |  | x |  |  |
| Spinal cord injury/ TM (yes/no) |  |  | x |  |  |
| Diagnostic testing |  |  | x |  |  |
| Classification |  |  | x |  |  |
| GBS (yes/no) |  |  | x |  |  |
| Diagnostic testing |  |  | x |  |  |
| GBS classification |  |  | x |  |  |
| Treatment |  |  | x |  |  |
| CIN/ CIM (yes/no) |  |  | x |  |  |
| CIN/ CIM criteria |  |  | x |  |  |
| Diagnostic testing |  |  | x |  |  |
| Treatment |  |  | x |  |  |
| Other neuropathy/ myopathy (yes/no) |  |  | x |  |  |
| Type |  |  | x |  |  |
| Hyposmia/ taste/ olfactory changes |  |  | x |  |  |
| Imaging (yes/no) |  |  | x |  |  |
| CT scan (head) |  |  | x |  |  |
| Report(s) |  |  | x |  |  |
| MRI (head/ spine) |  |  | x |  |  |
| Report(s) |  |  | x |  |  |
| Serum biochemistry (yes/no) |  |  | x |  |  |
| S100b |  |  | x |  |  |
| NSE |  |  | x |  |  |
| mRS at ICU discharge |  |  |  | x |  |
| mRS at 28-days after discharge |  |  |  | x |  |
| Withdrawal of life-saving therapies |  |  |  | x |  |
| Length of stay |  |  |  | x |  |
| Survival |  |  |  | x |  |
|  |  | | | | |

Table legend: mRS, modified Rankin Scale; ICH, intracranial hemorrhage; NIHSS, National Institute Health Stroke Score; GBS, Guillain Barré syndrome; CIN, critical illness neuropathy; CIM, critical illness myopathy; TM, transverse myelitis; CT, computed tomography; MRI, magnetic resonance imaging; CSF, cerebrospinal fluid.
